# Tractatus de Vermibus intestinorum, et Ictero

**Published:** 1783

**Authors:** 


V. Antonii de Haen *Tractatus de Vermibus inteſtinorum, et Ictero*; *edidit* F. de Waſſerberg. 8vo. Viennæ, 1780. p. 156.

THESE two tracts are offered to the public as part of a larger work ſince publiſhed in ſix volumes, 8vo. containing De Haen's Pathological Lectures on Boerhaave's Inſtitutes. The editor has every where interſperſed, in a parentheſis, his own obſervations and thoſe of the lateſt writers on the two ſubjects, ſo that his additions, eſpecially in the treatiſe on jaundice, are equal in bulk to the original text, and the whole may be conſidered as a pretty compleat collection of every thing that has been ſaid on the diſeaſes in queſtion.

Speaking of the tænia, the author mentions a ſingular caſe of a man, who, after labouring under a variety of diſtreſſing ſymptoms for many years, for which different remedies were preſcribed by his phyſician without effect, at length applied to a quack, who promiſed to cure him if he would ſwallow a certain quantity of ſoot every morning faſting. The remedy was taken, and produced at firſt nauſea, and an inſupportable uneaſineſs at his ſtomach; and ſoon afterwards a titillation in his fauces, from whence, with his fingers, he drew out a worm twenty ells long, and from that moment recovered his health.

Haller, in his edition of Boerhaave's Inſtitutes, makes him ſay that a Ruſſian, after taking *vitriolum martis*, voided a tænia 300 ells long; our author ſeems to hint, that Haller has fallen into a miſtake on this ſubject, as he never heard Boerhaave ſpecify the length of the worm;—"Ego vero—ſays he—nunquam audivi Boerhaavium definientem longitudinis vermis."

In children, whoſe complaints have originated from worms, he has often experienced the good effects of filings of ſteel, given every morning faſting, varying the doſe from five to twenty grains, according to the age of the patient. In

ſuch

ſuch caſes likewiſe, he has found the following formula ſingularly efficacious:

℞. Rad. phu,

—— filicis maris,

Polyp. querc.

Fol. Alexandrin. āā ℥ſs (i.e. Fol. Sennæ)

Sem. ſanton. ʒij.

Infunde cum cereviſiæ pinta. Sumat, pro ratione ætatis, jejunus omni die per dies 14, dein autem omni alterno die, ℥j, ij, iij, iv.

The author's ſhare of the treatiſe on jaundice conſiſts almoſt wholly of a commentary on the 773d paragraph of Boerhaave's Inſtitutions, where he ſays, *Bilis excretio nimia per ſuperiora, vel inferiora, orbat chylopoieſin primario inſtrumento.* The editor has added a good account of the late experiments and obſervations relative to the chemical properties of the bile, from M. Cadet and others; and likewiſe of a theory of jaundice, lately publiſhed by Dr. Marcard, who aſcribes it to a tranſudation of bile through the coats of the gall-bladder into the cavity of the abdomen, where it is taken up by the abſorbents.

In treating of biliary concretions, Dr. de Haen mentions the caſe of a woman who was under his care five years for a variety of complaints, but who had never any appearance of jaundice. After her death, upwards of a thousand small calculi were found in the gall-bladder.

